# Lateral position: a friendly surgical position for intramedullary nailing of tibial shaft fractures via infrapatellar approach

**DOI:** 10.1186/s12891-020-03883-1

**Published:** 2021-01-06

**Authors:** Jinzhu Zhao, Liang Qu, Peng Li, Changlong Tan, Chunsheng Tao

**Affiliations:** Department of Orthopedics, No.971 Hospital of the PLA Navy, 22 Ming-Jiang Road, 266071 Qingdao city, PR China

**Keywords:** Tibial shaft fracture, Intramedullary nailing, Surgical position, Infrapatellar approach, Lateral position

## Abstract

**Background:**

The conventional infrapatellar approach to intramedullary nailing of tibial fractures adopts the supine high-flexion knee position. However, this has disadvantages including difficulty in obtaining the proximal tibial anteroposterior view during intraoperative fluoroscopy, prolonged duration of fluoroscopy. Accordingly, the present study investigated the utility of the lateral position in the infrapatellar approach to intramedullary nailing of tibial shaft fractures.

**Methods:**

The present study was a retrospective analysis of 112 patients who sustained closed tibial shaft fractures and treated with intramedullary nailing via the infrapatellar approach. Patients were divided into two groups according to surgical position: lateral or supine. The demographic and clinical data were collected and analyzed.

**Results:**

There were 54 patients in the lateral and 58 in the supine position groups. The duration of surgery and fluoroscopy was shorter in the lateral group than the supine group (*p* < 0.05). Blood loss during surgery was lower in the lateral compared with supine position group (*p* < 0.05). The malunion rate was lower in the lateral position group as compared with the supine position group (*p* < 0.05); moreover, fewer surgical assistants were needed than in the supine group (*p* < 0.05). There were no significant differences in fracture healing time, other complications between the two groups (*p* > 0.05).

**Conclusions:**

The lateral position was a more convenient choice for intramedullary nailing of tibial shaft fractures via infrapatellar approach.

## Background

Tibial shaft fracture is the most common type of long tubular fracture[1]. For surgical treatment of closed tibial shaft fractures, intramedullary nailing is the first-line surgical option[2–5]. Intramedullary nailing has the advantages of reliable fixation, the possibility of earlier weight bearing, and less interference with soft tissues. In recent years, many studies have investigated various surgical approaches to and complications of intramedullary nailing[6–11]. A literature review suggested that the supine position has generally been adopted for the infrapatellar approach to intramedullary nailing of tibial shaft fractures; however, there are few, if any, reports describing the lateral supine position being used in intramedullary nailing.

Surgical position of the patient is highly important in terms of exposure and procedural mechanics. Proper positioning can facilitate the procedure and exposure, shorten operation time, and improve treatment efficiency. In recent years, significant advances have been made in intramedullary nailing of tibial shaft fractures in the semi-extended position using a suprapatellar portal technique, making it the more recommended method [7, 9, 12, 13]. The suprapatellar approach has certain advantages over the conventional infrapatellar approach, including a more comfortable position, better reduction of proximal fractures, shorter operation time and lower occurrence of anterior knee pain, among others [12, 14]. However, special surgical portal technology, tools, and longer learning curves are needed in the suprapatellar approach. In particular, interference with the patellofemoral joints[13] and the difficulty of internal fixation removal are disadvantages of this method[8]. Therefore, the infrapatellar approach remains the most familiar and common surgical approach used by most orthopedic surgeons. Nevertheless, supine with the knee hyperflexed is necessary during tibial intramedullary nailing using the infrapatellar approach. This position has shortcomings including difficulty in obtaining the proximal tibial anteroposterior view during intraoperative fluoroscopy, easy loss of alignment after fracture reduction, and prolonged duration of fluoroscopy[15].

Besides these shortcomings of the supine position that mentioned above, if no tool or postural frame is used, an extra assistant is often needed to hold and keep the knee flexed during infrapatellar nail insertion in supine position. Consequently, we have attempted to adopt the lateral position when performing intramedullary tibial nailing via the infrapatellar approach, and identified certain advantages over the conventional supine position. Therefore, the aim of this retrospective study was to investigate the utility of the lateral position in intramedullary nailing of tibial shaft fractures using the infrapatellar approach.

## Methods

### Patients

**Fig. 1:**
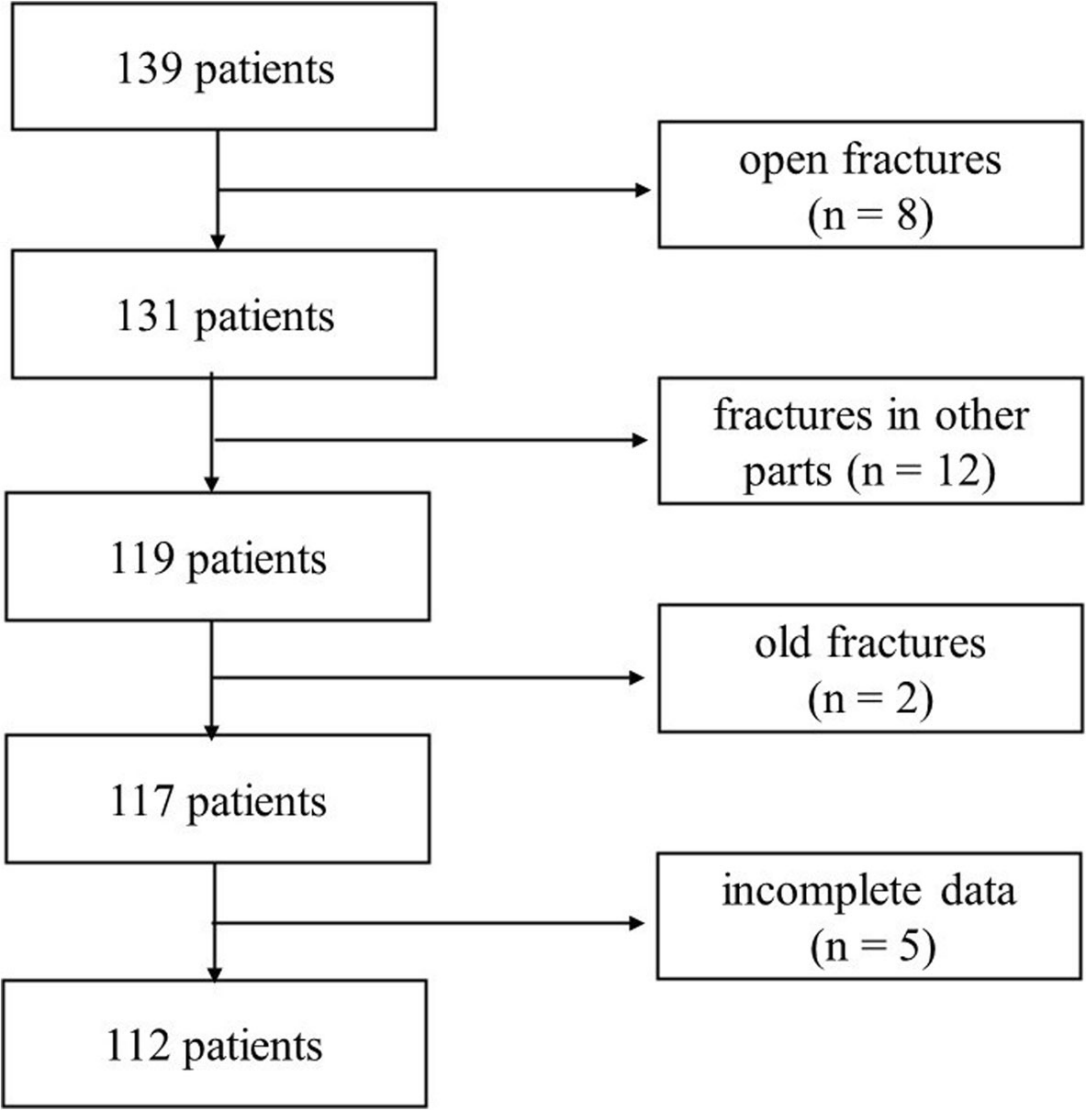
Selection of patients for the study

Data from patients with tibial shaft fractures, who underwent intramedullary nailing via the infrapatellar approach in the authors’ ward between January 2016 and January 2018, were retrospectively analyzed. Inclusion criteria were as follows: fresh, unilateral closed leg fracture; normal walking ability before fracture; and > 18 years of age with complete data. Individuals with old, pathological or open fractures, multiple fractures with breakage in other parts, no normal walking ability before the fracture, < 18 years of age, and those with incomplete follow-up data were excluded. Fractures were classified according to the AO Foundation/Orthopaedic Trauma Association (AO/OTA) fracture classification criteria. Patients were divided into one of two groups according to their positioning in surgery: supine and lateral. During the two-year period, 139 patients underwent intramedullary nailing for tibial fractures. After further analysis of case information, a total of 27 cases were excluded for the following reasons: open fracture(*n* = 8); fractures in other parts (*n* = 12); old fracture (*n* = 2); and incomplete data (*n* = 5). As such, a total of 112 patients were included in the present study (Fig. 1). All patients provided informed written consent for treatment. Given the retrospective design of the study and the use of anonymized patient data, the Ethics Committee of our hospital concluded that no approval of the committee was necessary.

### Surgical methods

Patients in both groups were treated with epidural anesthesia, and a tourniquet was secured on the fractured limb as backup. Tourniquets were not used for intramedullary nailing but were used when the fibular fracture plate was fixed. The infrapatellar approach and reamed interlocking intramedullary nailing technique was used for all patients. Fibular fracture was fixed in cases involving associated fracture in the distal-third of the fibula. The surgery was performed by the same surgeons.

### Lateral position group

**Fig. 2:**
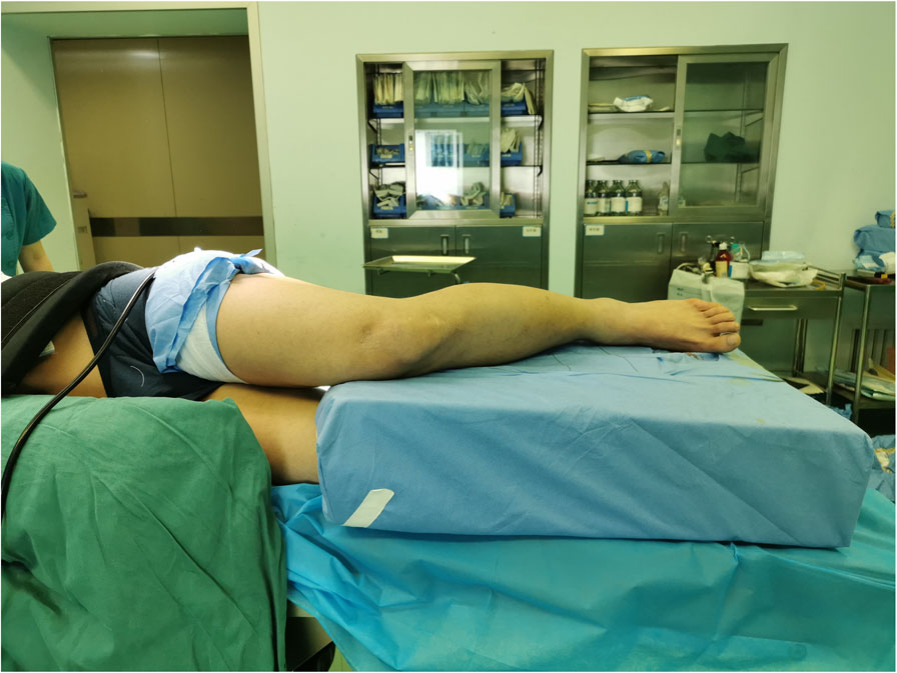
Clinical picture of the intra-operative lateral positiong

**Fig. 3:**
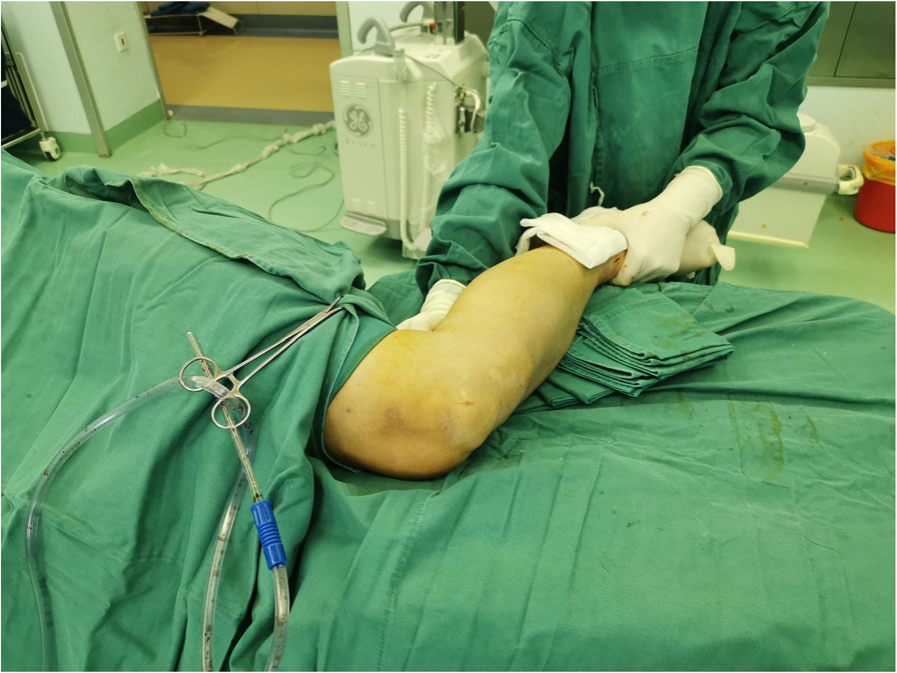
Assistant’s position during fracture reduction

**Fig. 4:**
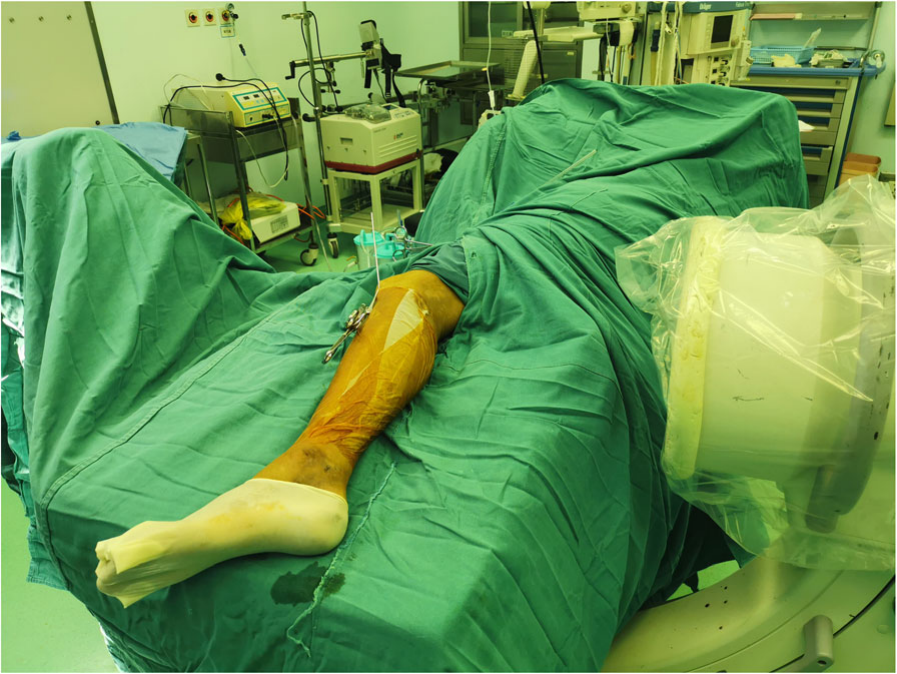
Proximal tibial anteroposterior view during intraoperative fluoroscopy

**Fig. 5:**
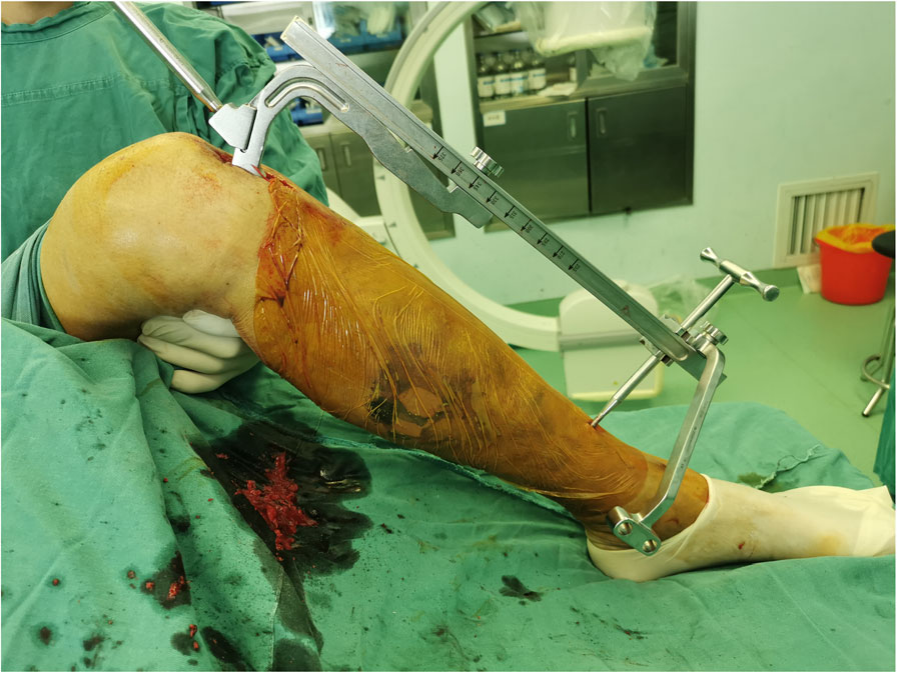
In case of distal and proximal locking, a shift of posture is required in lateral position

Patients were positioned laterally with the fractured limb placed on a custom-fabricated sponge cushion for the lateral lying position (as shown in Figs. 2). The surgeon is standing in front of the knee and the assistant is standing on the opposite side. During reduction, the knee was flexed to 90 degrees, the assistant holds distal femur with one hand and pulls the ankle with the other for countertraction (as shown in Figs. 3). The surgeon carries out a closed reduction of lateral displacement, angular displacement and rotatory displacement. Fluoroscopy of the proximal tibia, fracture and ankle should be performed when determining the starting point of proximal tibia and confirming the position of guide wire, respectively (as shown in Figs. 4). The knee can be kept at any position within the range of 10–120 degrees in this procedure. After insert the intramedullary nail, due to the impact of the proximal device against the patella, the knee is limited to a range greater than 90 degrees. In this case, the hip should be flexed by 90 degrees to perform a successful fluoroscopy. In type C and partial Type B fractures or some obese patients, the reduction of fractures maybe difficult, and a second assistant is needed who standing at the end of the operating table to assist in reduction. In the case of distal and proximal locking, a shift of posture is required in lateral position. Improper operation in this process can result in two serious problems: first, the loss of fracture reduction, especially the loss of rotatory reduction; second, the impact between the proximal device and the lower pole of the patella can result in patellar fracture and ligament injury. In order to avoid the two adverse results that mentioned above, there are two steps we should obey. Firstly, insert the intramedullary nail to the ideal depth in the lateral position. And then flex the hip and knee to 90 degrees while externally rotate the limb, in this procedure the assistant holds the knee with one hand and pulls the ankle appropriately with the other hand for resistance traction (as shown in Figs. 5). Secondly, after the position change, the operator reconfirms the reduction of the fracture by touching the anatomic marks of the tibial anterior crest. This is followed by distal and proximal locking in turn. By this method, we can avoid the loss of rotation reduction and the impact between the proximal device and the patella.

### Supine position group

The conventional supine position was adopted for this group. An additional assistant was required to elevate and fix the distal femur intraoperatively to keep knee flexion. And the other procedures were as usual as standard operation.

### Perioperative management

An intravenous drip of 1.0 g of cefazolin sodium was administered 30 min before surgery to prevent infection; the postoperative protocol included 200 mg of celecoxib per day (administered orally) for analgesia. X-ray films were re-examined on postoperative day 3 to assess fracture alignment and the position of the internal fixation. Regular outpatient review was performed at 1, 3, 6, and 12 months postoperatively using X-ray examination. Partial weight-bearing walking was started 6 weeks after the operation, and X-rays were reviewed during the follow-up period. Full weight-bearing started after the fracture healed.

### Data collection

Age, sex, body mass index, injury side, AO fracture type, injury mechanism, smoking history, diabetes, associated fibular fracture, fibula internal fixation, operation time, fluoroscopy time, intraoperative blood loss (determined by the assistant as the sum of gauze weighted plus the difference between the suction and irrigation volumes), number of assistants needed, complications (superficial infection, deep infection, malunion, delayed union, nonunion, anterior knee pain), fracture healing time, and the Lower Extremity Functional Scale (LEFT) score[16] at 12 months after surgery.

### Method to determine fracture healing

According to the radiographic union score for tibial (RUST) fractures[17], fracture healing is observed when osteotylus appears on 3 of the 4 cortical bones. Delayed union is defined as imaging not meeting healing standards at 6 months postoperatively, and nonunion is defined if imaging demonstrates no healing after 3 months of dynamic observation. Fracture healing was assessed using the standard method. Rotation deformity was based on the clinical evaluation method: knee flexion by 90° in prostrate position. The difference between the femoral-foot angle measurement of the fractured side and the healthy side was used to determine tibial rotation deformity. Varus and anteroposterior angle deformity were determined according to standard tibial orthotopic and lateral radiograph measurements. Clinically and, based on imaging results, any deformity > 10° is considered malunion.

### Statistical analysis

Statistical analysis was performed using SPSS version 19.00. The data were first tested for normality using the Kolmogorov-Smirnov method. Measurement data are expressed as mean ± standard deviation (SD), and count data are described as rates. Measurement data from the two groups were compared using the independent sample *t* test (normal distribution) or non-parametric H test (non-normal distribution), and the comparison of count data was performed using chi-squared analysis. Differences with *P* ≤ 0.05 were considered to be statistically significant.

## Results

There were 54 patients in the lateral and 58 in the supine position groups; demographic data of patients in the two groups are summarized in Table 1. There were no statistically significant differences in terms of age, sex, body mass index, fracture AO classification, trauma mechanism, smoking history, invovled limb, diabetes history and associated fibular fracture between the two groups (*p* > 0.05). And the pre-operation baseline conditions of the two groups were consistent.

**Table 1:** Demographic and baseline data of patients in the lateral and supine groups

Variable	Lateral group	Supine group
Age, years^a^	45.30(13.81)	45.02(12.62)
Sex (n, %) Male Female	42, 77.78% 12, 22.22%	47, 81.03% 11, 18.97%
BMI (kg/m2)^a^	24.44(2.67)	24.06(2.66)
AO/OTA classification (n, %) Type A Type B Type C	21, 38.89% 23, 42.59% 10, 18.52%	22, 37.93% 25, 43.10% 11, 18.97%
Trauma mechanism (n, %) Traffic accident Crushed Falling Sport injury Beaten	24, 44.44% 9, 16.67% 12, 22.22% 8, 14.81% 1, 1.85%	25, 43.10% 11, 18.97% 15, 25.86% 7, 12.07% 0, 0.00%
Involved limb (n, %) Left Right	22, 40.74% 32, 59.26%	22,37.93% 36, 62.07%
Smoking history (n, %)	16, 29.63%	19, 32.76%
Diabete history (n, %)	5, 9.26%	4, 6.90%
Associated fibular fracture (n, %)	54, 100%	58, 100%

^a^mean(SD); All *P*-value > 0.05

A comparison of surgical information and complications, fracture healing, and LEFT scores in the lateral and supine groups is presented in Table 2. The duration of the operation and the fluoroscopy time are significantly shorter in lateral position group, (72.98(14.21) minutes vs. 82.09(14.92) minutes, *p* = 0.001), (33.81(5.91) seconds vs. 68.53(8.69) seconds, *p* = 0.000), respectively. There was less blood loss during surgery in the lateral position group than in the supine group (95.65(14.51) ml vs. 121.90(44.54) ml, *p* = 0.000); the number of assistants required for surgery was smaller in the lateral group (*p* = 0.000); and the malunion rate was lower in the lateral group (*p* = 0.045). There was no significant difference in fracture healing time, infection or other complications, and LEFT scores at the 12 month follow-up examination (*p* > 0.05).

**Table 2:** Comparison of surgical data and complications, healing, and lower limb function scores in the lateral and supine groups

Variable	Lateral group	Supine group	*P*-value
Operation time (mintues)^a^	72.98(14.21)	82.09(14.92)	0.001
Fluoroscopy time (seconds)^a^	33.81(5.91)	68.53(8.69)	0.000
Intraoperative blood loss (milliliter)^a^	95.65(14.51)	121.90(44.54)	0.000
Number of assistants needed^a^	1.85(0.36)	2.43(0.50)	0.000
Fibula internal fixation (n,%)	29, 53.70%	30, 51.72%	0.293^*^
Complications (n,%) Superficial infection	2, 3.70%	2, 3.45%	0.994^*^
Deep infection	0, 0.00%	0, 0.00%	
Anterior knee pain	8, 14.81%	10, 17.24%	
Delayed fracture healing	3, 5.56%	2, 3.45%	
Nonunion	0, 0.00%	0, 0.00%	
Malunion (n,%) Rotation malunion Varus malunion Valgus malunion	3, 5.56% 1, 1.85% 0, 0.00%	10, 17.24% 1, 1.72% 0, 0.00%	0.045^*^
Time for union (weeks)^a^	14.00(5.93)	15.88(5.02)	0.072
LEFS score at 12 month^a^	77.19(2.39)	76.86(2.81)	0.515

^a^mean(SD); ^*^Chi-square-test

*LEFT *The Lower Extremity Functional Scale

## Discussion

Tibial shaft fracture is the most common type of long tubular bone fracture[1], and intramedullary nailing is currently the primary treatment of choice for most surgeons[2–5]. The main surgical approaches are infrapatellar and suprapatellar, which correspond to such surgical positions as supine with the knee hyperflexed and supine semi-extension. Surgical positions can have decisive impact on exposure and surgical flow, and can affect outcomes. An optimal surgical position facilitates surgical flow and exposure, shortens operation time, reduces bleeding, and protects the life of patients. There are some disadvantages to adopting the extreme knee flexion position when using the infrapatellar approach, including difficulty in observing the starting point during surgery, loss of fracture reduction, and long duration of fluoroscopy[15]. Although some investigators have attempted to solve these difficulties using special intraoperative traction devices[18], they often require additional traction equipment, increase the cost of surgery, and extend operation time, making it difficult for more widespread use. The present retrospective clinical study investigated the utility of the lateral position in intramedullary nailing of tibial shaft fractures through the infrapatellar approach.

We found that intramedullary nailing with the patient positioned laterally has many advantages. First, the knee joint can be flexed and extended through a wide range, which is conducive to intraoperative fluoroscopy and, especially, facilitates fluoroscopy of the proximal tibial nailing point and proximal fracture site. This is particularly important in lower-tier hospitals not equipped with orthopedic fluoroscopy beds, and can help solve the problem of ordinary surgical beds not being able to provide a complete anteroposterior view. Second, the rate of postoperative malunion in patients in the lateral position group was significantly lower than that in the supine group (p < 0.05). Analysis has revealed that the lateral position can reduce traction of the patellar tendon on the proximal tibia and the effect of gravity on the distal end of the fracture, thus helping to overcome shortcomings including difficulty of reduction with extreme knee flexion in the supine position and loss of reduction, thereby diminishing the possibility of postoperative malunion. This especially helps to lower the rate of malunion. Third, operation time, intraoperative fluoroscopy time, and intraoperative blood loss in the lateral position group were significantly lower than those in the supine group. This may be because the lateral position is more conductive to fluoroscopy and facilitates fracture reduction and maintenance, which will accelerate the operation and shorten the duration of intraoperative fluoroscopy and the surgery itself. In addition, internal plate fixation is required when tibia fractures are combined with fractures in the distal-third of the fibula. The lateral position is beneficial to surgical operation at the fibular fracture, and exposure of the surgical field is easier than in the supine position, which facilitates the involvement of assistants, which is another reason for the shorter operation time in the lateral position. In this retrospective study, we calculated intraoperative blood loss only. The shorter operation time in lateral position may lead to less intraoperative blood loss. However, further prospective controlled studies are needed to clarify whether there is a difference in postoperative hidden blood loss. Fourth, easier reduction of the fracture also means fewer surgical staff, helping to reduce the dependence on surgical assistants. The surgery can usually be completed with the help of one to two assistants, thereby reducing labor costs associated with the operation. Fifth, during the postoperative follow-up period, there was no significant difference between the two groups in the healing time of fractures, incidence of delayed healing or nonunion, or LEFT scores at the end of the follow-up period, suggesting that lateral positioning was not inferior to the conventional supine position in terms of early and mid-term treatment effects.

Despite these advantages, compared with the conventional supine position, the lateral position for intramedullary nailing also has some disadvantages, including a longer learning curve, the necessity for surgical assistants skilled and familiar with the operation, and the need for a well-coordinated team. These shortcomings, however, can be overcome by increased training for surgeons and assistants.

The present study had limitations, the first of which were its retrospective design and relatively small sample size. As such, studies with larger sample sizes will be needed to confirm our results. Furthermore, according to the results of this study, we speculate that the advantage of the lateral position in terms of fracture reduction may be more obvious in the intramedullary nailing of proximal tibial fractures, whereas in this study, cases of tibial shaft fractures were selected. Thus, further research investigating the utility of intramedullary nailing for proximal tibial fractures is warranted and may confirm the advantages of the lateral position.

## Conclusions

Tibial intramedullary nailing via the infrapatellar approach with patients in the lateral position overcomes the shortcomings of the conventional supine position, shortens the operation, the intraoperative use of fluoroscopy, and reduces the incidence of postoperative malunion, while also requiring no special tools or surgical instruments. Lateral supine is a more patient-friendly surgical position for fractures of the tibial shaft treated using intramedullary nailing via the infrapatellar approach.

## Data Availability

The datasets analyzed in the study are available from the corresponding author on reasonable request.

## Abbreviations

AO/OTA: AO Foundation/Orthopaedic Trauma Association
LEFT: Lower Extremity Functional Scale
